# Investing in a good pair of wellies: how do non-experts interpret the expert terminology of climate change impacts and adaptation?

**DOI:** 10.1007/s10584-019-02455-0

**Published:** 2019-06-17

**Authors:** Rachel Harcourt, Wändi Bruine de Bruin, Suraje Dessai, Andrea Taylor

**Affiliations:** 1 Univeristy of Leeds, Leeds, LS2 9JT UK; 2 Carnegie Mellon University, Pittsburgh, PA 15213 USA

**Keywords:** Climate change impacts, Adaptation, Science communication, Public engagement, Responsibility

## Abstract

The UK is already experiencing the impacts of climate change and these are expected to increase in scale and severity in the coming decades. Preparing for impacts by undertaking adaptive actions can potentially reduce the level of harm. In the UK, the government’s adaptation program aims to develop a “climate-ready society.” However, achieving broad public engagement in adaptation presents a significant communications challenge. Here, we aimed to understand how UK residents use and interpret the terms “climate change impacts” and “climate change adaptation.” We conducted a secondary analysis of 22 interviews with UK residents, who were recruited for their diverse climate change views. The interviewees expressed a lack of clarity around expected climate change impacts, which did not prevent them from saying that they were already experiencing the effects of a changing climate. Further, threats to cultural norms and values were perceived as serious and emotionally charged. Adaptation was often conflated with mitigation, and responsibility for adaptation was contested. We discuss the implications of our findings for developing more useful public communication about climate change adaptation.

**Electronic supplementary material:**

The online version of this article (10.1007/s10584-019-02455-0) contains supplementary material, which is available to authorized users.

## Introduction

### Communicating climate change in the UK

It is now accepted that due to past emissions of carbon dioxide and other greenhouse gases, some degree of climate change is inevitable (Moss et al. 2013; IPCC 2014a). A warming climate is expected to cause significant changes to the Earth’s systems bringing impacts such as more extreme weather patterns, rising sea levels, and increased drought (Houghton 2015). There is increasing scientific evidence that the effects of climate change are already being felt in the UK (Pall et al. 2011; Coumou and Rahmstorf 2012) and that they are expected to escalate over the coming decades (DEFRA 2012; CCC 2017). While the government’s UK Climate Change Risk Assessment has pointed to heavier rainfall, flooding, and heat waves as the most immediate threats, impacts to water resources, health, ecosystems, and agriculture are also possible (DEFRA 2012; CCC 2017). While some areas in the UK may be at more immediate risk than others, a changing climate in the UK is projected to eventually bring significant changes for all citizens (DEFRA 2012; CCC 2017).

Climate change adaptation aims to reduce the scale of harm from climate change impacts and maximize any benefits that arise (IPCC 2014b). The UK government has stated its ambition to become “a society which makes timely, far-sighted, and well-informed decisions” in adapting to climate change (DEFRA 2018, p. ii). But so far, only small steps have been taken to communicate this objective to UK residents. For example, the National Adaptation Programme, which is a publicly available parliamentary report that was first produced in 2013 and updated in 2018, details how the government intends to respond to the risks identified in the UK Climate Change Risk Assessment (DEFRA 2013, 2018, 2012; CCC 2017). This report recognizes that one of the government’s principle tasks will be to communicate to “deepen understanding” of risk and to “engage ever more people to take action” (DEFRA 2018, p.i). Additionally, long-running, national-scale risks, such as to domestic food production and international supply chains, will likely require communications that unite public support behind policy changes and financial investments (Bushell et al. 2015). Communications that make climate change risks and adaptive actions more tangible have been shown to increase public support for adaptation policies (Burch et al. 2013).

Nevertheless, compared with more established campaigns promoting mitigation and sustainability, the public communication of climate impacts and adaptation has been minimal (Ward 2014). So far, public communications have been borrowing climate experts’ terminology for “impacts” and “adaptation,” such as in the title of the National Adaptation Programme (DEFRA 2018). Perhaps, as a result, this specialized language is starting to creep into the broader public discourse, such as in this newspaper headline: “EU announces €9bn in funding for climate action… also pledging $300 million towards climate adaptation” (Harvey 2017). As the UK government seeks to increase public engagement in adaptation, it will need to develop communications which justify long-term financial and political investment (Adger et al. 2005). Additionally, as climate-related hazards increase, such as heat waves or flooding, public communications will need to provide advice on how individuals can act to minimize their exposure to negative harms. Hence, these communications would benefit from knowing how the concepts of climate change impacts and climate change adaptation are interpreted by non-expert audiences.

### Non-expert perception of climate change impacts

There is so far limited research on what UK residents know about climate change impacts that are likely to affect the UK (Taylor et al. 2014b). Available studies found that people were more likely to think of impacts, rather than causes or solutions, when asked to describe their associations of climate change or global warming (Lorenzoni et al. 2006; Smith and Joffe 2013). Associated impacts most frequently centered on extreme and negative weather patterns, particularly precipitation events and flooding (Lorenzoni et al. 2006; Whitmarsh 2009; Smith and Joffe 2013). However, UK residents also seemed to be less concerned about having more hot summers than about increases in flooding and rainfall (Taylor et al. 2014a). While UK residents tended to think of impacts to the UK, they also mentioned more distant impacts, such as melting ice (Smith and Joffe 2013).

However, in a UK-wide sample of 1800 interviewees, 69% were unsure of what climate change impacts will be and 40% questioned whether expert warnings of climate change seriousness have been over-emphasized (Poortinga et al. 2011). Skepticism towards impacts was much higher than skepticism towards warming trends or towards anthropogenic causes (Poortinga et al. 2011). Similarly, UK participants were unconvinced by attribution science, whereby climate experts try to establish to what extent unusual weather events can be attributed to anthropogenic climate change (Capstick and Pidgeon 2014b). Further, studies also found conflation between climate change and other environmental issues in the UK (Taylor et al. 2014b) and this misperception extended to understanding of impacts. For example, UK residents suggested air pollution and ozone depletion as possible outcomes of climate change (Lorenzoni et al. 2006; Smith and Joffe 2013).

While previous studies have asked participants about their associations of climate change which has led to the discussion of topics, such as extreme weather, which might be categorized by experts as impacts (e.g., Lorenzoni et al. 2006; Smith and Joffe 2013), studies have not previously focused on non-expert use and interpretation of the term climate change impacts.

### Non-expert understanding of climate change adaptation

The UK has been taking less adaptive action than its available resources and knowledge can facilitate (Tompkins et al. 2010; Ford et al. 2011). UK households also show a relatively low level of climate change adaptation (Porter et al. 2014). Generally, climate change adaptation remains low even amongst groups that are at risk of experiencing the negative effects of flooding (Whitmarsh 2008; Bichard and Kazmierczak 2012), sea level rise (Thomas et al. 2015; Bunyan et al. 2016), drought (Dessai and Sims 2010), and unusually cold winters (Capstick and Pidgeon 2014a). Often, people do not perceive the link between the extreme weather event and climate change (Whitmarsh 2008; Dessai and Sims 2010). Some studies have suggested that those who have experienced the negative effects of extreme weather still tend to think they are not at high risk of further threats (Bichard and Kazmierczak 2012; Thomas et al. 2015). Yet, others have shown that personal experience may increase the likelihood of taking actions to improve resilience to future threats (Spence et al. 2011; Demski et al. 2016).

It remains unclear how UK residents who do not perceive much risk of extreme weather use and interpret the concept of adaptation (Adger et al. 2017). UK residents seem to find it difficult to identify effective climate change adaptation actions. For example, they report short-term coping actions such as changing clothes in response to temperature extremes, rather than long-term adaptation, such as investing in home ventilation or insulation (Porter et al. 2014). Like Australians (Van Kasteren 2014), UK residents may confuse adaptive actions that aim to protect against impacts, with climate mitigation actions that aim to curb global warming.

Additionally, UK residents seem to doubt the efficacy of attempting to respond to climate change risks (Capstick and Pidgeon 2014b), although another study found support for preparing for a changing climate despite uncertainty about the risks (IPSOS MORI 2013). A further study found that disagreement as to whether the government or the individual was responsible for adaptation was one of the most divisive issues between the participants (Cotton and Stevens 2019). The limited number of studies available and the contrasting findings argue for the need for more research in this area.

### The current research

Our review of the literature finds that there are still only a few studies which research non-experts’ interpretation of climate change impacts and adaptation in the general population, as opposed to populations at high risk. Yet, if the UK government continues to develop an adaptation strategy for the country which promotes participation by all sectors of society, it will need to consider how to communicate adaptation so that a more diverse non-expert audience will feel that this is a relevant and important topic to address.

Here, we undertook a secondary analysis of interviews with UK residents with diverse climate change views. Our aim was to explore their use and interpretation of the terms impacts and adaptation. Our analysis of the data, therefore, focused on two research questions:

1. How do UK residents interpret the term climate change impacts?
2. How do UK residents interpret the concept of climate change adaptation?

## Method

### Data source

The secondary data analyzed in this research were interviews undertaken as part of the DEFRA-funded PREPARE (Programme of research on preparedness, adaptation and risk) project that examined UK public attitudes towards climate change impacts and preparations for climate risks (IPSOS MORI 2013). The program of work also comprised a nationally representative survey and a series of participatory workshops. The semi-structured interviews examined in this paper were conducted as a follow-up with a subset of survey participants with diverse views on climate change. The purpose of the interviews was to provide a further in-depth exploration of how the participants conceptualized adaptation.

Subsequent secondary analysis of the survey data showed that perceived increases in weather extremes were linked to stronger climate change beliefs (Taylor et al. 2014a) and that public adaptation priorities differed from those of experts (Taylor et al. 2017). Secondary analysis of the participatory workshops found that the participants tended to use morality-based arguments to support their opinions about adaptation (Adger et al. 2017). The interview data has not yet been analyzed in detail (IPSOS MORI 2013). The original research project was approved by the University of Leeds ethics committee and the interviews used here were approved for subsequent analysis.

### Sample

The interviews aimed to explore the use and interpretation of impacts and adaptation by individuals with a diverse set of climate change views. The interviewees were therefore selected based on views expressed in the previously completed survey (IPSOS MORI 2013; Taylor et al. 2014a). Table 1 lists the survey questions used as indicators of climate change views and the italicized text shows the responses selected for interview. The initial set of interviews (*n* = 15) was undertaken in March–April 2013. The second set (*n* = 7) was conducted in June–July 2014 with four repeat and three new interviewees. This sample size should be sufficient to uncover the most common ideas about impacts and adaptation, because the identification of new ideas tends to fall away after the first 10–15 interviews (Morgan et al. 2002). A diverse sample, as used here, also increases the likelihood of capturing a fuller range of ideas even when interviewing a relatively small number of people (Bruine De Bruin and Bostrom 2013). Demographic information is available in the Online Supplementary Material.

**Table 1:** Table of indicators used for interviewee selection. The 12 survey questions and answers listed in the table were used to indicate diverse climate change views. The responses shown in italics on the table were included in the final interviewee sample. The numbers in brackets show how all interviewees answered the questions

Indicators	Responses
Is climate change one of the three most important concerns for the UK?	*Yes (1)* No (16)
How convinced are you that climate change is currently affecting the UK?	*Totally convinced (4)* Fairly convinced (9) Not very convinced (1) Not at all convinced (2) Do not know (1)
Do you think humans have the right to modify the natural environment to suit their needs?	Strongly agree (1) Tend to agree (3) Neither agree or disagree (3) Tend to disagree (4) *Strongly disagree (5)* Do not know (1)
When I hear about climate change I feel:	*Worried (8)* *Sad (5)* *Angry (4)* Indifferent (4) Interested (4) Pessimistic (4) *Scared (3)* Happy (1) Optimistic (1)
During your lifetime, have you seen changes in UK weather?	*Yes, definitely (8)* Yes, probably (7) *Definitely not (1)* Do not know (1)
Have you or someone close to use experienced extreme weather events?	Flooding in the home (3) *Flooding in the local area (5)* Water restrictions or shortages (8) *Heat waves causing physical discomfort (8)* Heat waves disrupting travel (3) Heat waves affecting health (2) *No, none (5)*
During your lifetime, do you think the frequency of flooding has increased?	*A lot more frequently (9)* A little more frequently (6)
Do you expect your home to be at increased risk of flooding by 2050?	*Yes, definitely (2)* Yes, probably (2) *No, probably not (4)* No, definitely not (7) Do not know (2)
Do you have household insurance that covers for flooding and other climate change impacts?	*Definitely (8)* I think so (2) I’m not sure (2) No (5)
How likely do you think it is that heat waves will be more common in the UK by 2050?	Virtually certain (1) Likely (2) About as likely as not (5) Unlikely (7) Very unlikely (1) *Do not know (1)*
Who do you think is responsible for taking action to deal with the consequences of climate change?	*National government (15)* *Individuals and households (15)* Local authorities (12) Industry and business (11) Local communities (8) Environmental charities (3) Insurance companies (2)
What is the most important principle to consider when deciding how to respond to climate change impacts?	Protecting particularly vulnerable people such as the elderly and poor (9) *Minimizing the overall number of people at risk (4)* Avoiding loss of human life (2) Minimizing cost to business (1) Safeguarding our wildlife/landscape (1)

### Interview protocol

The semi-structured interviews built on the mental models approach which aims to characterize interviewee’s knowledge, as well as the language they choose to talk about it (Bruine De Bruin and Bostrom 2013). Mental models interviews provide useful insight for communications development because they can provide guidance on what topics or sub-topics need to be addressed by communications, as well as insight into the language and terminology currently being used to describe the topic by non-experts (Bruine De Bruin and Bostrom 2013). This information might not be accessible through surveys which necessarily provide some information to the participant, for example, in the wording of the questions, while often providing limited space for the participant to raise their own ideas. Mental models interviews have been gainfully used in previous research which similarly took an exploratory approach to understanding how certain ideas are conceptualized (Downs et al. 2008; Fizer et al. 2018).

Mental models interviews start with open-ended questions that allow interviewees to explain their views in their own words (Morgan et al. 2002). Here, interviews began by asking questions such as “Which three climate change impacts are you most worried about?” and “What three words or phrases come to mind when you think about climate change adaptation?” and then encouraged further exploration. Interviewees were also asked questions about the impacts they found most concerning, how the threat of climate change impacts made them feel, who they considered to be responsible for leading adaptation, and their own engagement with adaptation. The interview protocol is available in the Online Supplementary Material.

### Data analysis

In our secondary analysis of the interview transcripts, we aimed to explore the interviewees use and interpretation of the terms “climate change impacts” and “climate change adaptation” as they had been used in conversations about preparing for climate change in the UK. As such, the interviews were coded for the expression of ideas associated with those terms. The codes were developed inductively in line with our explorative approach to using this data (Thomas 2006). However, we intended to capture interpretations, for example, what actions the interviewees suggested as adaptation. Additionally, we wanted to capture how the interviewees used the terms in conversation and the extent to which they found them accessible, or not. The codes were used to develop a thematic analysis of the most frequently reoccurring ideas. The coding was done using qualitative analysis software.

## How do people living in the UK interpret the term climate change impacts?

### Climate impacts in the UK

Interviewees’ strongest association with the phrase climate impacts was extreme weather. This included heavy rain and flooding, hotter weather and water shortages, and storms, as well as seasons shifting their time through the year and/or becoming less clearly defined. Interviewees also suggested system-scale impacts including loss of wildlife and/or ecosystems, changes to the coasts through flooding and/or erosion, and impacts to farming and food production. Economic concerns, including the cost of living, more expensive or lack of access to insurance, and negative economic impacts on certain areas and industries such as the coasts and tourism, were mentioned. While the discussion principally focused on the UK, interviewees also showed awareness of global scale impacts including migration, food and water shortages, and global conflict. Climate change impacts were nearly always considered to be negative.

With the exceptions of one interviewee who reported a flooded garden and one who had bought ceiling fans during a hot spell, none of the interviewees had experienced the effects of extreme weather to their homes. Interviewees had a generally low sense of risk to themselves and their homes: “It’s not going to affect me personally… but I see it on the news” (female, 22). Justifications for this assessment included individual factors such as geographic location and/or economic position, as well as a sense that really dangerous levels of climate change impacts were a concern for some time in the future. Interviewees viewed the elderly, the very young, and nature as being more vulnerable than themselves.

More extreme dangers were expected to happen sometime in the future. Extreme weather and seasonal unreliability were expected to accelerate:

we’re going to get worse winters… summers, I think, where they alternate drought and flood, and it will be every year… if it isn’t flooding, it’s snowing, and if it isn’t snowing, we’ll be plunging down to minus 20…I personally think we’re on a slippery slope, climate-wise, unfortunately (male, 21)

There was an expectation that, at their most extreme, impacts would have catastrophic consequences, although this was not expected to occur until much further in the future: “we’re going to end up with just one man and an island in a thousand years from now!” (female, 32). However, the general agreement that things were definitely going to get worse was voiced alongside a perception that the future was largely unknowable: “2050, nobody knows! If anybody says they are just guessing” (male, 60). Even the scientists and experts “won’t really know what to expect, because they don’t know what’s happened. It’s never happened before” (female, 70).

### Experiencing climate change impacts

Nevertheless, interviewees also identified ways in which they felt that they, and the UK, were already experiencing the impacts of climate change. Increased disruption to daily life caused by more frequent and extreme weather was a reoccurring topic, as in the following:

the amount of times the M62 is gridlocked or snowed in, or closed, or something, which, once a year you can cope with but when it becomes an almost regular event, then it’s something else entirely… it just makes ‘day to day’ living more difficult (male, 35).

Negatively disruptive impacts covered a wide scale: individual dangers such as walking on icy pavements and driving on potholed roads; small scale inconveniences such as unexpected school closures; larger inconveniences such as failures of infrastructure; localized economic concerns such as the financial impact of flooding on homes, local businesses, and local industries such as tourism; and nationally shared concerns such as the rising cost of living driven by fuel and/or food costs.

Closely linked to the idea of more frequent disruptions, interviewees also referred to an increased sense of uncertainty. Again, this was usually related to extreme weather, such as this interviewee talking about heavy snow: “Obviously it doesn’t seem to be when it’s supposed to be… it always seems to be when you’re least expecting it” (female, 22). The uncertainty itself threatened to cause further daily disruption as people were unsure of how to adapt to the unexpected events. The interviewee above continued by saying that she works at the bingo where unexpectedly severe weather can negatively impact visitor numbers as customers cannot be sure if they will be able to travel home safely afterwards.

Additionally, interviewees thought that seasonal and annual weather patterns were becoming increasingly uncertain:

I don’t know whether it’s just the rose-coloured spectacles when you’re looking back, but you kind of knew your seasons, and they were always defined, and it was never extreme in any way, if you know what I mean, whereas now… it seems a bit more unpredictable and you never know quite what’s going to happen (male, 35)

As with the short but extreme weather events, this sense of seasonal uncertainty caused further disruption to daily life. For example, one interviewee suggested that it might be harder to plan for the school summer holidays which had previously been more reliably warm and sunny.

The use of “rose-colored spectacles” in the above quote suggests that the changes were being compared unfavorably with the way things were, or were remembered as being. It was an expression also used by others, as in this description of perceived seasonal change, “I remember sort of like proper winters and proper summers. Maybe I’m looking at it through rose-tinted glasses” (female, 54), as well as by a keen gardener referring to a recent lack of visiting wildlife compared with earlier years. The idea that climate change impacts in the UK would mean loss of the way things were was expressed in relation to a number of topics. These included seasonally based traditions that previous generations had enjoyed but were now unable to pass on to younger ones:

the girls having young children, and the weather as it is… I want them to be outside, want them to be playing… I can remember being a child and having weeks and weeks and weeks of nice, not changeable weather, but it seems to be changing all the while now (male, 49)

we certainly used to get the sledge out most years. My grandson has only used the sledge twice and he is four. It is a bit of a worry there isn’t it (female, 59)

Impacts to Britain’s natural scenery were also described in terms of loss. As with seasonal uncertainty, the interviewees commented both on the physical thing being impacted and the consequential loss this would cause to them or others. Changes to the coastlines, for instance, were perceived as risking the loss of traditional trips to the seaside. Particularly, resonant was the idea of wildlife depletion especially as it was being witnessed in the garden. Five interviewees said they felt that they were already suffering from an unwanted depletion of birds:

We get less birds these days don’t we? Which is a real shame, and that’s a real big loss, because I like that. I like birdsong and you just don’t seem to get as much of it as you used to (female, 54)

The topic of nature loss raised particularly emotional responses with one interviewee even stating “I hate human beings. I hate them with abundance” (female, 59).

### Impacts discussion

In summary, the term climate change impacts was understood to be closely associated with more extreme weather; of representing low personal risk to the interviewees; likely to get much more serious in the future, but already causing some disruptions and losses to daily life in the UK. Previous studies also found that UK residents associated climate change impacts with extreme weather (Lorenzoni et al. 2006; Smith and Joffe 2013). Studies of risk perception of extreme weather hazards have found that people tend to perceive themselves as low risk (Bichard and Kazmierczak 2012). Similarly, the perception that climate change impacts are likely to get much worse for later generations has been noted in previous public opinion research (Thomas et al. 2015).

However, our findings add two key points. Firstly, despite the lack of clarity on what climate impacts might be and how they themselves might be affected, there was a general consensus that the UK is already, and will in the future, experience serious climate change impacts. Despite interviewees’ diverse views on climate change, only one was completely skeptical on this point in their interview. Previous research has found that there is a high level of uncertainty regarding expected impacts as well as significant skepticism towards the supposed seriousness of coming climate change impacts (Poortinga et al. 2011). Here, the lack of certainty did not preclude assessment of climate change impacts as serious and of concern.

Secondly, interviewees perceived themselves as already experiencing climate change impacts other than the immediate effects of extreme weather events. Interviewees mentioned disruptions to their daily routines, shifts in the seasons, and a decline in British wildlife, amongst others. When discussing these ideas, the responses were often notably emotional. Some responses mentioned the frustration and uncertainty of greater disruption. Others voiced a sense of nostalgia towards unwanted changes from past times, and even loss. This tended to be expressed in relation to cultural values and traditions, such as weather-dependent traditions. It has been previously argued that accounting for values is necessary in identifying the motivations and limits of adaptive capacity within different communities (Adger et al. 2009; O'Brien and Wolf 2010). There is some evidence that the emotional resonance of values loss is already being utilized in some climate communications. Local newspapers, for instance, tend to use some signifiers of British values, such as changes to the local landscape and the quintessential English garden, as a means of increasing relevance and interest in their global climate change coverage (Brown et al. 2011). Further, there have been arguments for wide dissemination of a constructed narrative based on shared national values so as to motivate unified climate action (Bushell et al. 2015). However, so far, work on understanding what the most resonant national values might be and how they might be more deliberately utilized in public communication has not been developed. This research provides some preliminary ideas, for example, the emotional resonance of climate change impacts to wildlife and the seasons might be because these issues reflect key cultural touchpoints within British culture. Gardening is considered the national UK pastime while televised nature programs such as Spring Watch are regularly amongst the most watched (Lawrence 2009). Clearly, further targeted work using a larger sample is required and might likely reveal a segmentation of values which could inform the development of a range of communications. The limitations of a one-size-fits-all approach to communications which does not sufficiently speak to people’s real-life experiences and concerns have been well documented (Bostrom et al. 2013; Whitmarsh and Corner 2017). Additionally, while there is some evidence that framing climate change messages in terms of loss makes the information more memorable (Spence and Pidgeon 2010), overly negative messaging can be demotivating (O'Neill and Nicholson-Cole 2009). Any communications developed on the premise of promoting adaptation as a means to prevent loss of what we value would first need to be tested. But the importance attached to certain elements of British culture identified here offers a good starting point.

## How do people living in the UK interpret the term climate change adaptation?

### Conflation with mitigation

Interviewees associated the phrase climate change adaptation with ideas such as preparing, readying, and making changes. There was an assumption that the risks from climate change and its impacts made responding in some way inevitable: “the way I see it is that we’re going to have to adapt in terms of the weather’s going to get a lot worse” (female, 22). However, the range of ideas raised as to how this might be done signaled a more general understanding of the term adaptation. Some actions, such as building flood defenses or insulating the home, mostly aligned with the expert definition of adaptation as making adjustments to reduce harm from actual or expected climate. However, interviewees also discussed actions such as driving less, reducing domestic energy use, and recycling more, actions which would be categorized by experts as climate change mitigation and resource recovery. Interviewees talked about making lifestyle changes, such as being less materialistic, and attitude changes, such as being more aware of environmental impacts. There was also some mention of coping actions (planning for road closures) and of hazard avoidance (moving away from flood risk areas).

### Responsibility for adaptation

Interviewees principally saw instigating adaptation as the responsibility of their government. Reasons for this were the need for leadership, education, and information, although challenges such as increased taxes, short election cycles and public unwillingness towards a more controlling state were also raised. Other organized groups that were suggested to lead were charities, private businesses, and international governance bodies such as the United Nations. In all instances, reasons were provided as to why each group might fail to effectively tackle the challenge: it is too much for charities; private businesses are driven by financial interest; and international governance has too many parties and other issues to convene.

Individuals were also identified as having a role to play, although this was nearly always caveated with concerns. For example, individual action was thought to be unlikely due to lack of information or direction:

We all get told about how we can cut our carbon footprint and all the rest of that malarkey which as you say, just hopefully will stop it getting worse, but there’s not really been anything about, well, this is what you need to do. I presume if you live in Cornwall, you get a free supply of sandbags so they can adapt that way! (male, 35)

When asked to consider what they would do in a weather emergency, interviewees described the perceived lack of knowledge as a worrying concern and a barrier to action:

What me personally? ... I don't really know. A lot of the places where all the flooding happened I have no idea what those poor people are going to do… That would be a big worry (female, 62)

Wear warmer jackets!.. I don’t know what you would do about that but insulate your house properly. If you live in a flood plain I’m not quite sure what you do... You just have to make the best of it (female, 54)

Additionally, the likelihood of individual action being taken was caveated by a suggested lack of willingness of others to do their bit and/or concerns of limited individual impact. Despite this, the interviewees emphasized that they would be willing to undertake adaptive actions. It was not uncommon for concerns about individual capacity to act and personal willingness to be combined, as in below:

Interviewee: I’d like to be able to do something about it but I don’t know what and I don’t know how. It does make me worry, as I think, well, I try and do my bit. What are next door doing?

Interviewer: So, you’re ready, able and willing, as it were, but you need direction in what to do?

Interviewee: Yes.

(female, 32)

Nearly all interviewees suggested ways they currently were or would in the future take actions to adapt to climate change. As with the general discussion of adaptation, interviewees most frequently mentioned recycling and reduced use of fossil fuels in the home and the car. Interviewees use of the term adaptation suggested it was not necessarily understood as a strategy which could reduce their own risk from weather and climate-related harms. Instead, the term was often interpreted more broadly as a way to categorize all actions that respond to the challenge of climate change. One interviewee expressed this by first suggesting that he could adapt to climate change by recycling more. When asked if there is anything he could do to adapt to flooding or sea level rise, he at first hesitated, saying he did not think there was a simple solution and that he did not really know of anything individuals could do. When asked what he would do, he replied: “How I use power. Trying to make sure that your home’s insulated. Use the energy saving light bulbs, try and make sure your heating’s efficient, things like that” (male, 49). The interviewee went on to explain his response by saying that he can only manage how his home is run but he cannot control how the country is run. Whereas expert literature tends to categorize climate change action in terms of adaptation and mitigation, here the interviewee’s choice of actions seemed to be more influenced by his perception of the divide between individual and government responsibility.

One interviewee was asked if there was anything that they could or should do to adapt to climate change and they replied “Investing in a good pair of wellies!” (female, 22). She then laughed and said she was struggling to think, before suggesting recycling and driving less. The interviewer then asked her about extreme weather and whether there was anything she could do other than buy wellies and she replied: “Oh gosh, I can’t think of anything. I would only think in terms of keeping warm and things like that, and safe.” This quote exemplifies the willingness to engage with responding to climate change which many of the interviewees expressed. However, it also exemplifies the perceived lack of knowledge frequently raised during the discussions, as well as the tendency to understand the phrase “adapting to climate change” in more general terms to include mitigation responses. When asked specifically about extreme weather, the interviewee knows she wants to stay safe but says she cannot think of how she could do that. This suggests that not only the terminology but also the concept that it is possible to take adaptive actions to reduce personal risks from climate-related impacts is not yet well known.

### Adaptation discussion

To summarize, we find that when asked about adaptation, the interviewees suggested a range of ideas and that there was a general willingness to make changes in response to climate change. However, when interviewees were pushed on responding to the idea of adapting to extreme weather and other climate hazards, responses were much more hesitant and unsure. Specifically, understanding of adaptation as taking preparatory actions so as to reduce harm should climate-related events occur was low. Interviewees generally struggled to think of adaptive actions that they or others could undertake in response to specific risks such as flooding.

These findings rise two important learnings for communicators. Firstly, the term adaptation seems to evoke interpretations that differ from those used by experts. For example, as with earlier research done in other countries (e.g., Van Kasteren 2014), this UK sample frequently conflated adaptation with mitigation. Adaptation is a term in general use and, therefore, it is understandable that people might transpose their current usage of the word on to this specialist field. Here, interviewees suggested recycling more and driving less as ways of adapting to climate change, for instance. This is a fair response to the question while at the same time focusing on actions which experts would not define as adaptive, and that would not necessarily help someone prepare for climate-related hazards. So far, publicly available government messaging is replicating the expert terminology, as in the UK's National Adaptation Programme. These findings suggest that this terminology assumes a specific definition that is not necessarily shared by the targeted audience.

Secondly, the appropriate location of responsibility for adaptation was a contested question, as found in other UK studies (Cotton and Stevens 2019). Interviewees tended to think that individuals were at least partially responsible for adapting. Further, interviewees said that they were themselves currently taking adaptive actions or would be willing to in the future. However, despite this general sense of willingness, both the appropriateness and feasibility of individual responsibility was questioned. Additionally, the issue of not knowing what to do was raised as a significant barrier to taking action. This was particularly the case when the questions focused in on how to respond to extreme weather events, as opposed to how to adapt to climate change. One of the key findings coming through from adaptation research in developed nations so far is the extent to which adaptation taking place is much less than would be possible based on available knowledge and resources (Ford et al. 2011). This is the case even in households which have previously been impacted (Porter et al. 2014) or are at higher risk of imminent negative impacts (Bichard and Kazmierczak 2012). The reasons for this can be multiple and complex and not entirely dependent on whether the individuals have sufficient information or not (Toole et al. 2016; Bichard and Kazmierczak 2012). Perception of individual adaptive capacity, i.e., that possible adaptive actions are considered to be doable and affordable, can act as a barrier or motivator, for instance (Grothmann and Patt 2005; Van Valkengoed and Steg 2019). The potential gap between intentions expressed during an interview and actions taken in real life also needs to be noted in relation to these findings. However, these findings suggest that there remains a preliminary information deficit about actions which individuals can take to manage the risks from climate-related hazards.

## Limitations

Undertaking inductive secondary analysis of existing data will unavoidably mean that the areas identified as of interest will differ from the original intentions of the research (Adger et al. 2017). Our focus on the interpretation of expert terminology is well informed by the existing data but would have further benefited from targeted interview topics and questions. For example, it would have been insightful to learn if the interviewees took adaptive actions which they did not necessarily recognize as such and so did not talk about in these interviews. The interviews provided rich, in-depth detail and relevant insight regarding the governance of adaptation in the UK. However, a larger, cross-national survey could provide insight into the prevalence of specific interpretations and associations will willingness to act (Bruine De Bruin and Bostrom 2013).

## Conclusion

As a changing climate brings more and wider reaching impacts to the UK, there is a need to increase public support to develop a “climate-ready society.” An adaptation communications campaign will need to address a complex and potentially worrying subject while being informative and engaging for those most acutely at risk, as well as those providing support for national-scale readying.

Here, we have reported on the findings from a secondary analysis of interviews undertaken with UK residents holding diverse climate change views to capture their understanding of the terms “climate change impacts” and “climate change adaptation.” The principle findings are that expected climate change impacts cover a large range and scale but are nevertheless seen as already happening and likely to increase over the coming decades. This is perceived as concerning, with impacts to cultural values and traditions being particularly emotionally charged. The term adaptation, as it is used by climate change experts, is not a familiar concept and is often conflated with mitigation. Perceived location of responsibility for adaptation is also unclear.

These findings raise both warnings and potential benefits for adaptation communicators. The use of expert terminology risks reducing the accuracy and saliency of the messaging. Communicators may need to either choose other language from outside of the expert dialog or more actively communicate what the expert terms mean as a preliminary to further information/engagement messaging. However, these findings also suggest that there are some shared cultural values which might provide a way of increasing the perceived relevance and seriousness of impacts and adaptation, particularly useful when targeting an audience with mixed climate change views and diverse levels of personal risk.

Future research should provide a more comprehensive study into how impacts and adaptation are being narrated in all sectors of the public discourse. This research has argued that so far, official communications are borrowing the expert terminology to talk about impacts and adaptation. However, the general public get much of their climate change information from other non-official sources of communication, such as the media. Understanding how other contributors to the public discourse are talking about adaptation, in terms of both content and language choices, is a necessary further insight needed for the development of effective public communications.

## Electronic supplementary material

ESM 1 (DOCX 29 kb)

## References

[CR1] Adger WN, Arnell NW, Tompkins EL (2005). Successful adaptation to climate change across scales. Glob Environ Chang.

[CR2] Adger WN, Dessai S, Goulden M, Hulme M, Lorenzoni I, Nelson DR, Naess LO, Wolf J, Wreford A (2009). Are there social limits to adaptation to climate change?. Clim Chang.

[CR3] Adger WN, Butler C, Walker-Springett K (2017). Moral reasoning in adaptation to climate change. Enviromental Politics.

[CR4] Bichard E, Kazmierczak A (2012). Are homeowners willing to adapt to and mitigate the effects of climate change?. Clim Chang.

[CR5] Bostrom A, Bohm G, O’Connor RE (2013). Targeting and tailoring climate change communications. Wiley Interdiscip Rev Clim Chang.

[CR6] Brown T, Budd L, Bell M, Rendell H (2011). The local impact of global climate change: reporting on landscape transformation and threatened identity in the English regional newspaper press. Public Underst Sci.

[CR7] Bruine De Bruin W, Bostrom A (2013). Assessing what to address in science communication. PNAS.

[CR8] Bunyan S, Collins A, Duffy D (2016). Concern and helplessness: citizens’ assessments of individual and collective action on the provision of environmental public goods in a coastal city at risk of inundation. Environ Manag.

[CR9] Burch SLM, Sheppard SRJ, Pond E, Schroth O, Moser SC, Boykoff MT (2013). Climate change visioning: effective processes for advancing the policy and practice of local adaptation. Successful adaptation to climate change: linking science and policy in a rapidly changing world.

[CR10] Bushell S, Colley T, Workman M (2015). A unified narrative for climate change. Nat Clim Chang.

[CR11] Capstick SB, Pidgeon NF (2014). Public perception of cold weather events as evidence for and against climate change. Clim Chang.

[CR12] Capstick SB, Pidgeon NF (2014). What is climate change scepticism? Examination of the concept using a mixed methods study of the UK public. Global Environmental Change-Human and Policy Dimensions.

[CR13] CCC 2017 UK climate change risk assessment 2017 Evidence report key messages from the synthesis report [Online]. Available: https://www.theccc.org.uk/wp-content/uploads/2016/07/CCRA-Synthesis-Report-Key-Messages-fact-sheet-1.pdf [Accessed 6th February 2017]

[CR14] Cotton M, Stevens E (2019). Mapping discourses of climate change adaptation in the United Kingdom. Weather, Climate, and Society.

[CR15] Coumou D, Rahmstorf S (2012). A decade of weather extremes. Nat Clim Chang.

[CR16] DEFRA (2012) Summary of the key findings from the UK climate change risk assessment 2012 [Online]. Available: http://randd.defra.gov.uk/Document.aspx?Document=Summary_of_Key_Findings.pdf [Accessed 14th October 2017]

[CR17] DEFRA (2013) The national adaptation programme: making the country resilient to a changing climate [Online]. Available: https://www.gov.uk/government/uploads/system/uploads/attachment_data/file/209866/pb13942-nap-20130701.pdf [Accessed 10th January 2017]

[CR18] DEFRA (2018) The national adaptation programme and the third strategy for climate adaptation reporting: making the country resilient to a changing climate [Online]. Available: https://assets.publishing.service.gov.uk/government/uploads/system/uploads/attachment_data/file/727252/national-adaptation-programme-2018.pdf [Accessed 23rd July 2018]

[CR19] Demski C, Capstick S, Pidgeon N, Gennaro Sposato R, Spence A (2016). Experience of extreme weather affects climate change mitigation and adaptation responses. Clim Chang.

[CR20] Dessai S, Sims C (2010). Public perception of drought and climate change in Southeast England. Environ Hazard.

[CR21] Downs JS, DE Bruin WB, Fischhoff B (2008). Parents’ vaccination comprehension and decisions. Vaccine.

[CR22] Fizer C, Bruine De Bruin W, Stillo F (2018) Barriers to managing private wells and septic systems in underserved communities: mental models of homeowner decision making. J Environ Health:81

[CR23] Ford JD, Berrang-Ford L, Paterson J (2011). A systematic review of observed climate change adaptation in developed nations: a letter. Clim Chang.

[CR24] Grothmann T, Patt A (2005). Adaptive capacity and human cognition: the process of individual adaptation to climate change. Glob Environ Chang.

[CR25] Harvey F (2017) EU announces €9bn in funding for climate action [Online]. Available: https://www.theguardian.com/environment/2017/dec/12/eu-announces-9bn-in-funding-for-climate-action [Accessed 10th May 2018]

[CR26] Houghton JT (2015). Global warming: the complete briefing.

[CR27] IPCC (2014a) Climate change 2014 impacts, adaptation, and vulnerability: working group Ii contribution to the fifth assessment report of the intergovernmental panel on climate change [Online]. Available: https://www.ipcc.ch/report/ar5/wg2/ [Accessed 12th April 2017]

[CR28] IPCC (2014b) IPCC fifth assessment report working group II contribution: glossary [Online]. Available: http://www.ipcc.ch/report/ar5/wg2/ [Accessed 11th September 2017]

[CR29] IPSOS MORI (2013) PREPARE - climate risk acceptability findings from a series of deliberative workshops and online survey [online]. Available: http://randd.defra.gov.uk/Document.aspx?Document=11261_PREPARECA0513Publicclimateriskacceptability-Finalreport.pdf [Accessed 15th October 2017]

[CR30] Lawrence A (2009). The first cuckoo in winter: phenology, recording, credibility and meaning in Britain. Glob Environ Chang.

[CR31] Lorenzoni I, Leiserowitz A, De Franca Doria M, Poortinga W, Pidgeon NF (2006). Cross-national comparisons of image associations with “global warming” and “climate change” among laypeople in the United States of America and Great Britain 1. J Risk Res.

[CR32] Morgan MG, Fischoff B, Bostrom A, Atman C (2002). Risk communication: a mental models approach.

[CR33] Moss RH, Meehl GA, Lemos MC, Smith JB, Arnold JR, Arnott JC, Behar D, Brasseur GP, Broomell SB, Busalacchi AJ, Dessai S, Ebi KL, Edmonds JA, Furlow J, Goddard L, Hartmann HC, Hurrell JW, Katzenberger JW, Liverman DM, Mote PW, Moser SC, Kumar A, Pulwarty RS, Seyller EA, Turner II BL, Washington WM, Wilbanks TJ (2013). Hell and high water: practice-relevant adaptation science. Policy Forum.

[CR34] O'Brien KL, Wolf J (2010). A values-based approach to vulnerability and adaptation to climate change. Wiley Interdiscip Rev Clim Chang.

[CR35] O'Neill S, Nicholson-Cole S (2009). “Fear won’t do it” promoting positive engagement with climate change through visual and iconic representations. Sci Commun.

[CR36] Pall P, Aina T, Stone DA, Stott PA, Nozawa T, Hilberts AGJ, Lohmann D, Allen MR (2011). Anthropogenic greenhouse gas contribution to flood risk in England and Wales in autumn 2000. Nature.

[CR37] Poortinga W, Spence A, Whitmarsh L, Capstick S, Pidgeon NF (2011). Uncertain climate: an investigation into public scepticism about anthropogenic climate change. Global Environmental Change-Human and Policy Dimensions.

[CR38] Porter JJ, Dessai S, Tompkins EL (2014). What do we know about UK household adaptation to climate change? A systematic review. Clim Chang.

[CR39] Smith N, Joffe H (2013). How the public engages with global warming: a social representations approach. Public Underst Sci.

[CR40] Spence A, Pidgeon N (2010). Framing and communicating climate change: the effects of distance and outcome frame manipulations. Glob Environ Chang.

[CR41] Spence A, Poortinga W, Butler C, Pidgeon NF (2011). Perceptions of climate change and willingness to save energy related to flood experience. Nat Clim Chang.

[CR42] Taylor A, Bruine De Bruin W, Dessai S (2014). Climate change beliefs and perceptions of weather-related changes in the United Kingdom. Risk Anal.

[CR43] Taylor AL, Dessai S, Bruine De Bruin W (2014). Public perception of climate risk and adaptation in the UK: a review of the literature. Clim Risk Manag.

[CR44] Taylor A, Bruine De Bruin W, Dessai S (2017) Public priorities and expectations of climate change impacts in the United Kingdom. J Risk Res

[CR45] Thomas DR (2006). A general inductive approach for analyzing qualitative evaluation data. Am J Eval.

[CR46] Thomas M, Pidgeon N, Whitmarsh L, Ballinger R (2015). Mental models of sea-level change: a mixed methods analysis on the Severn Estuary, UK. Glob Environ Chang.

[CR47] Tompkins EL, Adger WN, Boyd E, Nicholson-Cole S, Weatherhead K, Arnell N (2010). Observed adaptation to climate change: UK evidence of transition to a well-adapting society. Glob Environ Chang.

[CR48] Toole S, Klocker N, Head L (2016). Re-thinking climate change adaptation and capacities at the household scale. Clim Chang.

[CR49] Van Kasteren Y (2014). How are householders talking about climate change adaptation?. J Environ Psychol.

[CR50] Van Valkengoed AM, Steg L (2019) Meta-analyses of factors motivating climate change adaptation behaviour. Nat Clim Chang

[CR51] Ward B (2014) Climate adaptation must be backed by climate communication [online]. Available: https://www.businessgreen.com/bg/opinion/2356602/climate-adaptation-must-be-backed-by-communication [Accessed 17th April 2018]

[CR52] Whitmarsh L (2008). Are flood victims more concerned about climate change than other people? The role of direct experience in risk perception and behavioural response. J Risk Res.

[CR53] Whitmarsh L (2009). What’s in a name? Commonalities and differences in public understanding of “climate change” and “global warming”. Public Underst Sci.

[CR54] Whitmarsh L, Corner A (2017). Tools for a new climate conversation: a mixed-methods study of language for public engagement across the political spectrum. Global Environmental Change-Human And Policy Dimensions.

