# Supplementary material for: Investing in a good pair of wellies: how do non-experts interpret the expert terminology of climate change impacts and adaptation?
Source: Clim Change. 2019 Jun 17;155(2):257–72. doi: 10.1007/s10584-019-02455-0 (PMC6647866; doi:10.1007/s10584-019-02455-0)
Supplement: Supplementary file 1 — (DOCX 29 kb) [file 10584_2019_2455_MOESM1_ESM.docx]

**Appendix – Online Supplementary Material**

***Appendix 1 - Interview Protocol***

I would like you to think for a moment about the impacts of climate change in the UK. Could you tell me which are the three impacts you are most concerned about?

Prompt - Can you explain why these three are of particular concern to you?

Are there more impacts that concern you that we haven’t talked about?

(I can note these down and if we have time left at the end we can talk about them.) – use in question 4.

Now I would like you to think for a moment about adapting to climate change in the UK. What are the first three words or phrases that come into your mind?

Prompt - Can you explain why you mentioned those three things in particular?

Generally when I’m talking with someone about climate change, one of the main issues that always pops up in the conversation, is how individuals, businesses, organisations, and the Government, respond to extreme weather events, such as heavy rain or snow, gales, floods, heat waves, droughts, extremely low temperatures, etc. I am going to ask you your opinion on that later but firstly, can you describe to me your experiences of extreme weather?

Prompt - I can make a list and then ask for each one mentioned:

When was this?

What affect did it have on you?

What did you do?

Thinking back over your life in the UK, do you personally feel that over the long term you have seen any notable changes in the weather the UK experiences?

Earlier I asked you about the impacts of climate change in the UK.

Prompt - I can refer to the top three plus any others mentioned by the respondent

Are there any impacts of climate change that you think are particularly significant?

Prompt - To whom are they significant, you, your family and friends, specific organisations, everybody? What about wildlife and natural habitats?

Do any of these people, groups or habitats need extra protection from specific impacts?

Prompt – Are there groups that are more vulnerable such as the elderly, businesses, endangered species, others?

Which of the impacts you have expressed concern for do you think we will experience in the UK by 2050? (Repeat the list if necessary)

How does this make you feel?

Prompt - Perhaps you could describe for me any overall positive or negative outcomes you can foresee.

I mentioned earlier that we would have an opportunity to discuss responses to extreme weather events. If this is something we have to do as part of our adaptation to climate change, who do you think is responsible for leading on this?

Prompt - Are there different types of organisation or individual that should be involved?

Is there anything you can or should be doing to adapt to climate change?

Prompt – I can use the lists of impacts to probe for specific actions.

***Appendix 2 - Table of Demographic Information***

| **Interviewee Number** | **Gender** | **Age** | **Region** |
| --- | --- | --- | --- |
| 1 | Female | 22 | North West |
| 2 | Female | 32 | Wales |
| 3 | Female | 48 | North East |
| 4 | Female | 54 | Yorkshire & Humberside |
| 5 | Female | 59 | Yorkshire & Humberside |
| 6 | Female | 62 | Northern Ireland |
| 7 | Female | 70 | Wales |
| 8 | Male | 21 | Greater London |
| 9 | Male | 21 | North West |
| 10 | Male | 32 | North East |
| 11 | Male | 35 | North West |
| 12 | Male | 41 | South West |
| 13 | Male | 44 | Wales |
| 14 | Male | 49 | West Midlands |
| 15 | Male | 50 | West Midlands |
| 16 | Male | 60 | Scotland |
| 17 | Male | 66 | South East |
| 18 | Male | 70 | South East |
